# *Schistosoma mansoni*-Derived Lipids in Extracellular Vesicles: Potential Agonists for Eosinophillic Tissue Repair

**DOI:** 10.3389/fimmu.2019.01010

**Published:** 2019-05-07

**Authors:** Gillian Coakley, Mark D. Wright, Jessica G. Borger

**Affiliations:** Department of Immunology and Pathology, Central Clinical School, Monash University, Melbourne, VIC, Australia

**Keywords:** *S. mansoni*, eosinophil, TGFβ, lipid, extracellular vesicle, exosomes

The co-evolution of helminths with their hosts has required these parasites to develop a range of sophisticated molecular mechanisms and adaptations to evade, suppress and activate host cells to maximize survival and maintain infection within their chosen niche. Recent studies have revealed that *Schistosoma mansoni (S. mansoni)*-derived lipids are agonists of innate pattern recognition receptors on eosinophils, mediating a pro-fibrotic phenotype. Indeed, the release of lipids from *Schistosoma* could be a key factor driving disease pathogenesis in hepatosplenic forms of the infection, where excessive hepatic fibrosis is linked to significant morbidity. A fundamental question that remains is how are lipids derived from the tegumental outer surface of *S. mansoni* adult worms, cercariae and eggs, transported and protected from the inflammatory milieu to target and activate surface receptors on eosinophils. The recent identification of lipid-enriched extracellular vesicles (EVs) as an evolutionarily conserved form of host-pathogen communication, has led us to speculate that *S. mansoni*-derived extracellular vesicles are responsible for the targeting of bioactive lipids to eosinophils, and we argue that these cargo delivery systems may be an influential factor in both tissue repair and fibrosis during helminth infection.

## Schistosomal Lipids Trigger Eosinophilic Tissue Repair

Schistosomiasis is an infectious parasitic disease caused by the trematode flukes of the genus *Schistosoma*. The schistosmiasis lifecycle involves free-living larval cerceriae that penetrate human skin, mature into schistosomula in the tissues then migrate through the lungs and systemic circulation before residing in either vesical (S. *haematobium)* or mesenteric (*S. japonicum*, and *S. mansoni)* veins as sexually mature adults where they can evade the immune system for years (1). Females release eggs which either leave the body in excreta to propagate the lifecycle, or get trapped in the tissues inducing a granulomatous immune response. The schistosoma granuloma is a complex immune structure comprised of macrophages and eosinophils in a concentric inner ring with infiltrating dendritic cells and CD4^+^ T cells interspersed surrounded by an outer layer of fibroblasts which form around eggs deposited in the liver (2), which can result in clinical morbidity from the ongoing fibrosis that encases calcified eggs. In hepatosplenic schistosomiasis, chronic complications develop as a consequence to the inflammatory response to either *S. mansoni or S. japonicum*, causing excessive hepatic fibrosis which results in portal hypertension and congestive splenomegaly, the most common causes of mortality associated with this disease (3).

Eosinophillia is a prominent feature during *S. mansoni* infection, with exponential increases in the levels of eosinophils in peripheral blood correlating with disease progression and granuloma development within the host liver during acute infection. Eosinophils comprise ~44–60% of cells within the schistosoma granuloma during acute and chronic infection, respectively (4). Given the persistence of eosinophils during infection, their role in tissue remodeling and fibrosis during and/or following infection has been postulated, especially due to the association between chronic eosinophilia and other fibrotic conditions (5). The participation of eosinophils and type-2 immunity in tissue remodeling and repair was recently demonstrated in murine models of infection where eosinophils were shown to directly drive IL-4-mediated wound repair and regeneration as a post-toxin injury response in skeletal muscle and liver tissue (6, 7). Interestingly, eosinophil-derived IL-4 was shown to support *T. spiralis* new-born larval growth in muscle tissue by limiting the interferon-driven local inflammatory environment (8). However, type-2 cytokines are not the only molecules secreted by eosinophils to induce wound repair. Another pro-fibrotic mediator human eosinophils are known to release is Transforming Growth Factor-beta (TGF-β), which contributes strongly to airway remodeling in asthma (9), fibroblast proliferation and matrix deposition in the lung (10).

Whilst studies continue to define the role of eosinophils in tissue repair during helminth infection, what remains unexplored within current literature is how this occurs through recognition of parasite-derived pathogen associated molecular patterns. A new report by Magalhaes and colleagues has discovered that lipids derived from *S. mansoni*, namely lysophosphatidylcholine (LPC) and prostaglandin (PG)D2, can activate eosinophils via toll-like receptor 2 (TLR2) and prostaglandin D2 receptor 1 (DP1) promoting the release of TGF-β to support both fibrosis and tissue repair (11). This novel finding built upon the authors previous research that established LPC signaling through TLR2 mediates eosinophil recruitment and function during *S. mansoni* infection, whereby TLR2 deficient mice lacked strong type-2 immunity and blood/tissue eosinophilia (12). Interestingly, recent studies from the same group demonstrated a similar effect of schistosomal LPC on macrophages, in which macrophages polarized to an alternatively-activated M2 phenotype through a PPAR-γ-dependent mechanism and were capable of secreting TGF-β, identifying a common pathway to potentiate tissue repair in response to the recognition of schistosomal lipids within the microenvironment (13). It would be reasonable to speculate that the continual release of these active lipid mediators during chronic infection could be a major contributing factor in the excessive fibrotic response observed in hepatosplenic schistosomiasis, and an attractive target for therapeutic intervention.

Exactly how eosinophils and potentially macrophages and dendritic cells within the granuloma and the periphery receive lipid signals from *S. mansoni* is poorly understood. We argue that it is highly unlikely to only occur through direct contact with the egg or schistosomula due to limited mobility within the center of the granuloma, particularly after the onset of fibrosis. Furthermore, the majority of schistosomula die before sexual maturity, with this necrotic process compromising tegumental integrity and facilitating the rapid enzymatic destruction of lipid components. Together this would suggest that like chemical messengers, schistosomal lipids must be actively released into the granulomatous environment and mesenteric veins to access target cells in the microenvironment in a bioactively stable form. We propose a central role exists here for extracellular vesicles; highly lipid enriched messengers utilized by cells to transport proteins and nucleic acids to mediate cell: cell, and more recently, host: pathogen communication.

## Extracellular Vesicles as a Form Of Host: Pathogen Communication

The phospholipid LPC is highly surface active (44.3 dyn/cm) (14), so assembly of LPC within the lipid bilayer of the worm membrane could indeed have potent effects on those immune cells which come into direct contact as suggested by Magalhães et al. (11). It was proposed that LPC and other lysophospholipids may be excreted as degradation products of the worm tegument, activating the TLR2 pathway as apoptotic biproducts (12). However, tegumental phospholipids have been demonstrated to have a far shorter half-life than those contained within the worm body and LPC is rapidly metabolized by lysophospholipase and LPC-acyltransferase which instead, strongly suggests that unbound LPC would be immediately degraded *in vivo* following worm necrosis and have limited biological activity.

Lipids are poorly water soluble so need to act either in short range or be transported by specific carriers such as lipoproteins. Magalhães et al. use an artificial lipid worm extract in their studies representing a highly pure and concentrated helminth product not derived from a necrotic process, nor structurally contained within a tegumental lipid enriched bilayer (11). Interestingly, a recent *S. mansoni* lipidome study found LPC to be enriched in cercariae and eggs, although only present as a minor phospholipid in the adult worm suggesting the amount of LPC within the worm tegument may not be sufficient to engage and ligate TLRs to drive cellular activation (15). Furthermore, the importance of targeted delivery of LPC, which would not be achieved by the release of apoptotic bodies, is emphasized by the rapid degradation of lysophospholipids by lipid phosphate phosphatases present on the surface of all cells, enzymes shown to rapidly hydrolyze and reduce the effective local concentration of the lipid agonist (16). Thus, for LPC to interact with surface receptors on eosinophils *in vivo* it is highly likely that LPC from the worm tegument is concentrated and tightly packaged within the lipid bilayer of *S. mansoni*-derived extracellular vesicles and actively released.

Exosomes are submicron bioactive extracellular vesicles released through a regulated pathway from all healthy cells of the body as a mechanism of intercellular communication. In recent years, the definition of different forms of extracellular vesicles has become more defined owing to their ubiquity in many biological and disease contexts. As such, minimal guidelines have been introduced to classify different populations of extracellular vesicles, including exosomes, ectosomes, and microparticles (17). Within the parent cell, the molecular sorting of its cytosolic contents including proteins and nucleic acids into intra-luminal vesicles encased by a cholesterol-enriched lipid bilayer is regulated by intercellular RabGTPases. Exosomes have been shown to transport an array of GTP-activatable phospholipases and prostaglandins (PGs) packaged within the lipid bilayer from cell to cell (18) [reviewed in Sagini et al. (19)]. Due to the potential immunomodulatory nature of different types of extracellular vesicle, as was described in the adult liver fluke, *Fasciola hepatica* (20), we will refer to any potential vesicles secreted by *S. mansoni* as extracellular vesicles, that presumably may be of exosomal origin. Extracellular vesicles are released from the intracellular *Leishmania spp*. and *Trypanosoma cruzi* parasites as well as extracellular pathogens, providing a mechanism for the import of parasite cargo into host cells, including virulence factors from *Trichomonas vaginalis* and *Trypanosoma brucei* (21, 22). In regard to helminth infections, extracellular vesicles have been shown to be a common component in the excretory-secretory product (20, 23, 24). Recent investigations of *Echinostoma caproni, Fasciola hepatica, Dicrocoelium dendriticum, Schistsoma japonicum, Opisthorchis viverrine, Heligmosomoides polygyrus, and Trichuris suis* demonstrated that exosomes are excreted from helminths and can be taken up by immune cells (25–30) and notably *S. mansoni* and *S. japonicum* exosomes shown to transport potential host modulating proteins, miRNAs, and tsRNAs (23, 31). We first identified that *H. polygyrus* released extracellular vesicles that were present in the excretory-secretory product of the adult parasitic worm and revealed that these bioactive vesicles could alter host gene expression, suggesting extracellular vesicles are a highly specialized mechanism for shuttling parasite factors into host cells to modulate the immune system (32).

## *S. mansoni-derived* Extracellular Vesicles Deliver Lipid Agonists to Trigger TLRs on Eosinophils

Lipids are a critical component of exosomes and small extracellular vesicles, forming the protective lipid bilayer which is directly exposed to the environment and forms the interacting surface with recipient host cells. Surprisingly, the lipid content of helminth-derived exosomes remains relatively unknown, with only a small number of entries for vesicle cargo devoted to lipids in the online database Vesiclepedia. Of those published, the majority list the lipid composition of human-derived immune cells and cancer cell lines [reviewed in Yáñez-Mó et al. (33)]. The identification of the agonists of PGR and TLR2 receptors by Magalhães et al. suggests that lipids embedded within the membranous bilayer of extracellular vesicles secreted by helminths are more than just structural components and may indeed act as novel second messengers within the inflammatory environment (11).

It is well known that exosomes, in comparison to their cellular origin, are highly enriched in an array of lipid species, including phosphatidylserine, sphingomyelin, cholesterol, and plasmalogen. The composition of the lipid moiety within exosomes can not only influence their stability *in vivo*, but can also have their own functional consequences. Exosomes have been shown to interact with cell peripheral lipid receptors such as Tim4 which recognizes phosphatidylserine (34). Lyso-phosphatidylserine extracted from the tegument of *S. mansoni* has been shown to activate TLR2 and direct dendritic cell polarization. Fascinatingly, the effect of lyso-phosphatidylserine on TLR2 was specifically mediated by the parasite lyso-phosphatidylserine species as a commercial synthetic and mammalian-derived lyso-phosphatidylserine had no effect on TLR2 activation (35). Similarly, PGs have been identified in exosomes, with vesicular PGE2 enriched in T cell derived exosomes (36). A recent study of the *S. mansoni* lipidome found PDG2 to be the most abundant prostaglandin, identified in cercariae and eggs, and was particularly enriched in soluble egg antigen, worm secretory product and egg excretory/secretory product, strongly suggesting PGD2 is released in extracellular vesicles within the excretory/secretory product of *S. mansoni in vivo* (15). Thus, it is highly plausible that LPC and PG species in the lipid bilayer encasing *S. mansoni* derived-extracellular vesicle contents are themselves able to modulate the immune response upon recognition by surface receptors on target cells.

**Figure 1:**
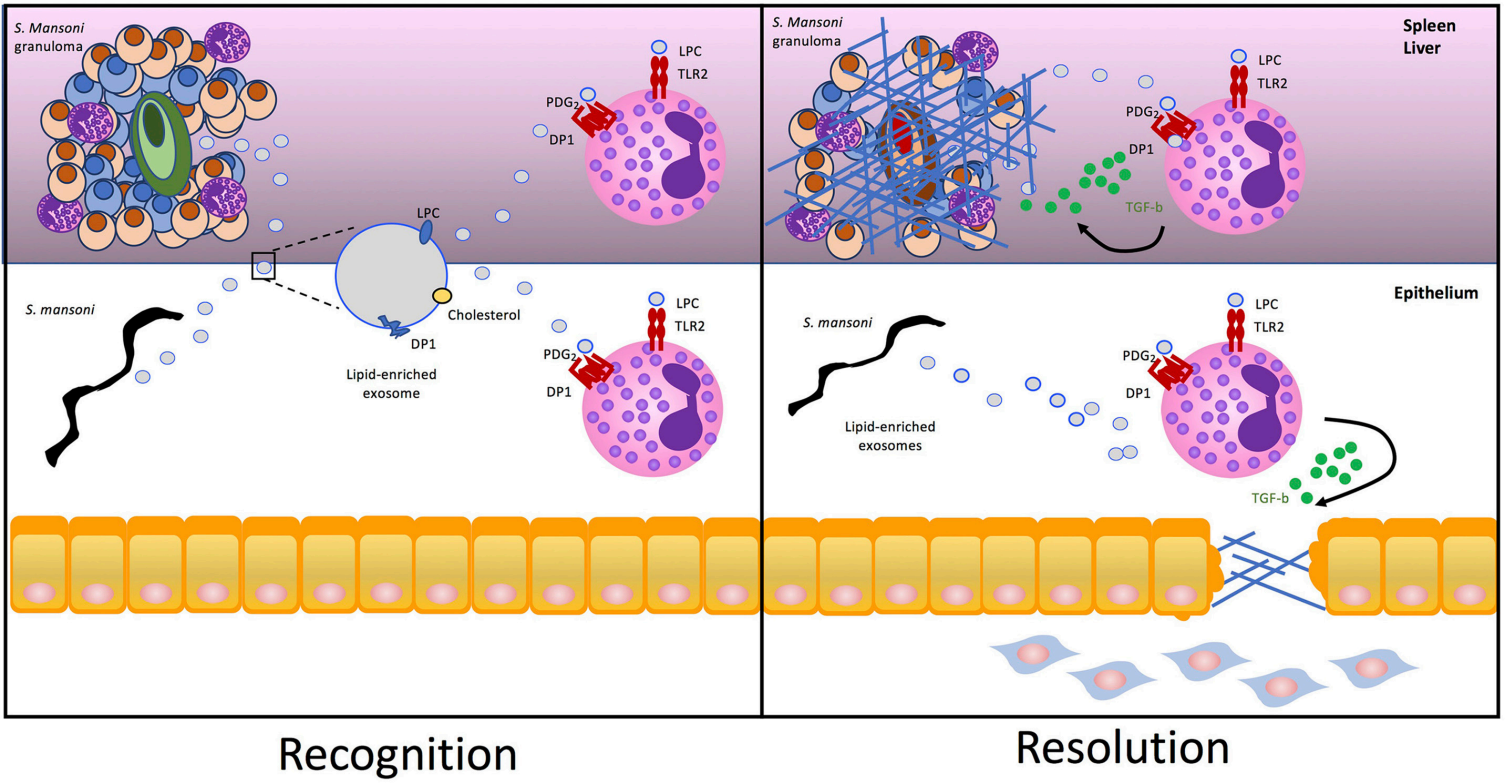
*S. mansoni* lipid-enriched extracellular vesicles trigger eosinophil tissue repair. *S. mansoni* eggs and worms release extracellular vesicles exosomes enriched in nucleic acid, proteins, cholesterol, and lipids including LPC and DP1. Packaging in the bilayer of exosomes protects lipids from enzymatic degradation once released in the inflammatory milieu as a component of the helminth excretory/secretory product allowing LPC and PGD2 to be targeted for delivery and recognition by TLR2 and PDG1, respectively, on the surface of eosinophils. Activation of TLR2 and DP1 by *S. mansoni*-derived exosomes drives lipid droplet accumulation within eosinophils and release of pro-fibrotic TGF-β to drive fibrosis in the granuloma or epithelium.

It has been postulated that exosome recognition by cells involves G2A, a G protein coupled receptor that recognizes LPC on the surface of exosomes (37). Autotaxin, the lysophospholipase responsible for generating lisophosphatidic acid (LPA) from its substrate LPC, is an enzyme which once secreted can bind to the surface of exosomes (38). Exosome-bound autotaxin is catalytically active and can bind to the host cell through specific integrin interactions, facilitating the release of LPA to activate cell surface G-protein-coupled receptors (38). It is tempting to speculate that a similar mechanism of action exists for the delivery and recognition of LPC by TLR2 on eosinophils. Therefore, we propose the mechanism underlying the findings of Magalhães et al. involves active release and targeted binding of *S. mansoni*-derived LPC-loaded extracellular vesicles to deliver the lipid agonist to TLR2 on eosinophils, and a similar exosomal delivery method of PDG2 may also exist (Figure 1).

## *S. mansoni* Extracellular Vesicles as Vaccine Candidates–Resetting the Balance Between Tissue Repair and Fibrosis

Complex participation from the different life stages of *S. mansoni* including cercariae, soluble egg antigen and even the sex of the adult worm can drive potent host immunosuppression (39, 40). As such, isolating helminth products with similar immunomodulatory properties, such as those found in parasite excretory-secretory products or from the parasite itself, may represent a target for vaccine development. Recent reports have highlighted the use of helminth-derived extracellular vesicles to prevent future infection. Indeed, we recently found that vaccination of mice with extracellular vesicles derived from *H. polygyrus* protected against a subsequent infection, inducing high titres of EV-specific antibodies (32). Similar immunomodulatory properties were demonstrated with *S. japonicum*-derived exosomes which were shown to induce M1 macrophage polarization (27). Vaccines have also been directed against specific molecules enriched in extracellular vesicles, such as cathepsins and heat-shock proteins, rather than the entire extracellular vesicles (41). Currently, *S. mansoni*-derived proteins are being tested as vaccines against Schistosomiasis with promising results (42), with the fatty-acid binding protein Sm14, which plays a role in *S. mansoni* lipid uptake, assortment, and transport, currently being trialed as a potential vaccine candidate in humans and animals (43). Although the lipids investigated in the recent study by Magalhães et al. are shown to potentiate a tissue repair cascade, chronic release of these mediators could potentially drive an unfavorable disease phenotype, characterized by advanced hepatic fibrosis, aggravated portal hypertension and the induction of splenomegaly. As such, targeting these lipid-vesicle complexes by vaccination could neutralize an excessive fibrotic response, whilst simultaneously directing immunity against parasite-derived lipids (especially if these same lipids can be found on a particular life-stage of the parasitic worm). Moreover, if the vaccine could prevent establishment of the parasite and perturb it's lifecycle, this would diminish subsequent inflammation and progression of severe hepatosplenic fibrosis, which is far beyond the reparative fibrosis of granulomas that occurs in patients who have the benign hepatointestinal form of the disease.

It is likely that vesicle secretion by the parasite, host or both is associated with co-evolutionary adaptations of both parasite and host alike to maintain a chronic infection whilst attempting to resolve damage to host tissues. Unfortunately, an excessive tissue-repair response may result in unfavorable clinical outcomes, such as hepatosplenic fibrosis. The identification of LPC and PDG2 as potential extracellular vesicle-derived targets (in which the mechanism of action has been established) highlights their potential clinical applications 2-fold. Enrichment of LPC and PDG2 lipids within the serum above levels of a healthy subject could be used as diagnostic biomarkers and it would be of interest to see if serum levels of these lipids correlated with disease severity or more excitingly, increased fibrosis. Moreover, LPC and PDG2 have been identified as promising new vaccine targets in masonic and japonica schistosomiasis. Now it is of importance to determine whether targeting these schistosomal lipids through vaccination will alter the recruitment and activation of eosinophils to the site of inflammation and influence or limit disease pathology during schistosome infection, which could have broader implications for infections in which eosinophils play a key role.

## Author Contributions

JB and GC drafted the manuscript. JB, GC, and MW edited the manuscript. JB, GC, and MW approved the final version.

### Conflict of Interest Statement

The authors declare that the research was conducted in the absence of any commercial or financial relationships that could be construed as a potential conflict of interest.

## References

[1] Colley DG Bustinduy AL Secor WE King CH. Human schistosomiasis. Lancet. (2014) 383:2253–64. 10.1016/S0140-6736(13)61949-2 24698483 PMC4672382

[2] Chuah C Jones MK Burke ML McManus DP Gobert GN. Cellular and chemokine-mediated regulation in schistosome-induced hepatic pathology. Trends Parasitol. (2014). 30:141–50. 10.1016/j.pt.2013.12.009 24433721

[3] Olveda DU Olveda RM McManus DP Cai P Chau TN Lam AK. The chronic enteropathogenic disease schistosomiasis. Int J Infect Dis. (2014) 28:193–203. 10.1016/j.ijid.2014.07.009 25250908

[4] Amaral KB Silva TP Dias FF Malta KK Rosa FM Costa-Neto SF. Histological assessment of granulomas in natural and experimental *Schistosoma mansoni* infections using whole slide imaging. PLoS ONE. (2017) 12:e0184696. 10.1371/journal.pone.0184696 28902908 PMC5597217

[5] Aceves SS. Remodeling and fibrosis in chronic eosinophil inflammation. Dig Dis. (2014) 32:15–21. 10.1159/000357004 24603375 PMC4037288

[6] Heredia JE Mukundan L Chen FM Mueller AA Deo RC Locksley RM. Type 2 innate signals stimulate fibro/adipogenic progenitors to facilitate muscle regeneration. Cell. (2013) 153:376–88. 10.1016/j.cell.2013.02.053 23582327 PMC3663598

[7] Goh YP Henderson NC Heredia JE Red Eagle A Odegaard JI Lehwald N. Eosinophils secrete IL-4 to facilitate liver regeneration. Proc Natl Acad Sci USA. (2013). 110:9914–9. 10.1073/pnas.1304046110 23716700 PMC3683773

[8] Huang L Beiting DP Gebreselassie NG Gagliardo LF Ruyechan MC Lee NA. Eosinophils and IL-4 support nematode growth coincident with an innate response to tissue injury. PLoS Pathog. (2015) 11:e1005347. 10.1371/journal.ppat.1005347 26720604 PMC4697774

[9] Minshall EM Leung DY Martin RJ Song YL Cameron L Ernst P. Eosinophil-associated TGF-beta1 mRNA expression and airways fibrosis in bronchial asthma. Am J Respir Cell Mol Biol. (1997). 17:326–33. 10.1165/ajrcmb.17.3.2733 9308919

[10] Levi-Schaffer F Garbuzenko E Rubin A Reich R Pickholz D Gillery P. Human eosinophils regulate human lung- and skin-derived fibroblast properties in vitro: a role for transforming growth factor beta (TGF-beta). Proc Natl Acad Sci USA. (1999) 96:9660–5. 10.1073/pnas.96.17.9660 10449750 PMC22266

[11] Magalhães KG Luna-Gomes T Mesquita-Santos F Corrêa R Assunção LS Atella GC. Schistosomal lipids activate human eosinophils via toll-like receptor 2 and PGD2 receptors: 15-LO role in cytokine secretion. Front Immunol. (2018) 9:3161. 10.3389/fimmu.2018.03161 30740113 PMC6355688

[12] Magalhães K Almeida PE Atella G Maya-Monteiro CM Castro-Faria-Neto H Pelajo-Machado M. Schistosomal-derived lysophosphatidylcholine are involved in eosinophil activation and recruitment through Toll-like receptor-2-dependent mechanisms. J Infect Dis. (2010). 202:1369–79. 10.1086/656477 20863227

[13] Assunção LS Magalhães KG Carneiro AB Molinaro R Almeida PE Atella GC. Schistosomal-derived lysophosphatidylcholine triggers M2 polarization of macrophages through PPARgamma dependent mechanisms. Biochim Biophys Acta Mol Cell Biol Lipids. (2017). 1862:246–54. 10.1016/j.bbalip.2016.11.006 27871882

[14] Munder PG Modolell M Andreesen R Weltzien HU Westphal O Lysophosphatidylcholine (lysolecithin) and its synthetic analogues. Immunemodulating and other biologic effects. Springer Semin Immunopathol. (1979) 2:187–203. 10.1007/BF01891668

[15] Giera M Kaisar MMM Derks RJE Steenvoorden E Kruize YCM Hokke CH. The *Schistosoma mansoni* lipidome: leads for immunomodulation. Anal Chim Acta. (2018). 1037:107–18. 10.1016/j.aca.2017.11.058 30292284

[16] Reue K Brindley DN. Thematic review series: glycerolipids. Multiple roles for lipins/phosphatidate phosphatase enzymes in lipid metabolism. J Lipid Res. (2008) 49:2493–503. 10.1194/jlr.R800019-JLR200 18791037 PMC2582367

[17] Théry C Witwer KW Aikawa E Alcaraz MJ Anderson JD Andriantsitohaina R. Minimal information for studies of extracellular vesicles 2018 (MISEV2018): a position statement of the International Society for Extracellular Vesicles and update of the MISEV2014 guidelines. J Extracell Vesicles. (2018) 7:1535750. 10.1080/20013078.2018.1535750 30637094 PMC6322352

[18] Buratta S Urbanelli L Sagini K Giovagnoli S Caponi S Fioretto D. Extracellular vesicles released by fibroblasts undergoing H-Ras induced senescence show changes in lipid profile. PLoS ONE. (2017) 12:e0188840. 10.1371/journal.pone.0188840 29182668 PMC5705128

[19] Sagini K Costanzi E Emiliani C Buratta S Urbanelli L. Extracellular vesicles as conveyors of membrane-derived bioactive lipids in immune system. Int J Mol Sci. (2018) 19:1227. 10.3390/ijms19041227 29670015 PMC5979532

[20] Cwiklinski K de la Torre-Escudero E Trelis M Bernal D Dufresne PJ Brennan GP. The extracellular vesicles of the helminth pathogen, *Fasciola hepatica*: biogenesis pathways and cargo molecules involved in parasite pathogenesis. Mol Cell Proteomics. (2015) 14:3258–73. 10.1074/mcp.M115.053934 26486420 PMC4762619

[21] Silverman JM Clos J Horakova E Wang AY Wiesgigl M Kelly I. Leishmania exosomes modulate innate and adaptive immune responses through effects on monocytes and dendritic cells. J Immunol. (2010) 185:5011–22. 10.4049/jimmunol.1000541 20881185

[22] Szempruch AJ Sykes SE Kieft R Dennison L Becker AC Gartrell A. Extracellular vesicles from *Trypanosoma brucei* mediate virulence factor transfer and cause host anemia. Cell. (2016) 164:246–57. 10.1016/j.cell.2015.11.051 26771494 PMC4715261

[23] Nowacki FC Swain MT Klychnikov OI Niazi U Ivens A Quintana JF. Protein and small non-coding RNA-enriched extracellular vesicles are released by the pathogenic blood fluke *Schistosoma mansoni*. J Extracell Vesicles. (2015) 4:28665. 10.3402/jev.v4.28665 26443722 PMC4595467

[24] Zamanian M Fraser LM Agbedanu PN Harischandra H Moorhead AR Day TA. Release of small RNA-containing exosome-like vesicles from the human filarial parasite *Brugia malayi*. PLoS Negl Trop Dis. (2015) 9:e0004069. 10.1371/journal.pntd.0004069 26401956 PMC4581865

[25] Marcilla A Trelis M Cortés A Sotillo J Cantalapiedra F Minguez MT. Extracellular vesicles from parasitic helminths contain specific excretory/secretory proteins and are internalized in intestinal host cells. PLoS ONE. (2012) 7:e45974. 10.1371/journal.pone.0045974 23029346 PMC3454434

[26] Bernal D Trelis M Montaner S Cantalapiedra F Galiano A Hackenberg M. Surface analysis of *Dicrocoelium dendriticum*. The molecular characterization of exosomes reveals the presence of miRNAs. J Proteomics. (2014) 105:232–41. 10.1016/j.jprot.2014.02.012 24561797

[27] Wang L Li Z Shen J Liu Z Liang J Wu X. Exosome-like vesicles derived by *Schistosoma japonicum* adult worms mediates M1 type immune- activity of macrophage. Parasitol Res. (2015) 114:1865–73. 10.1007/s00436-015-4373-7 25855345

[28] Chaiyadet S Sotillo J Smout M Cantacessi C Jones MK Johnson MS. Carcinogenic Liver fluke secretes extracellular vesicles that promote cholangiocytes to adopt a tumorigenic phenotype. J Infect Dis. (2015) 212:1636–45. 10.1093/infdis/jiv291 25985904 PMC4621255

[29] Hansen EP Kringel H Williams AR Nejsum P. Secretion of RNA-containing extracellular vesicles by the porcine whipworm, *Trichuris suis*. J Parasitol. (2015) 101:336–40. 10.1645/14-714.1 25723295

[30] Buck AH Coakley G Simbari F McSorley HJ Quintana JF Le Bihan T. Exosomes secreted by nematode parasites transfer small RNAs to mammalian cells and modulate innate immunity. Nat Commun. (2014) 5:5488. 10.1038/ncomms6488 25421927 PMC4263141

[31] Samoil V Dagenais M Ganapathy V Aldridge J Glebov A Jardim A. Vesicle-based secretion in schistosomes: analysis of protein and microRNA (miRNA) content of exosome-like vesicles derived from *Schistosoma mansoni*. Sci Rep. (2018) 8:3286. 10.1038/s41598-018-21587-4 29459722 PMC5818524

[32] Coakley G McCaskill JL Borger JG Simbari F Robertson E Millar M. Extracellular vesicles from a helminth parasite suppress macrophage activation and constitute an effective vaccine for protective immunity. Cell Rep. (2017) 19:1545–57. 10.1016/j.celrep.2017.05.001 28538175 PMC5457486

[33] Yáñez-Mó M Siljander PR Andreu Z Zavec AB Borràs FE Buzas EI. Biological properties of extracellular vesicles and their physiological functions. J Extracell Vesicles. (2015) 4:27066. 10.3402/jev.v4.27066 25979354 PMC4433489

[34] Zakharova L Svetlova M Fomina AF. T cell exosomes induce cholesterol accumulation in human monocytes via phosphatidylserine receptor. J Cell Physiol. (2007) 212:174–81. 10.1002/jcp.21013 17299798

[35] van der Kleij D Latz E Brouwers JF Kruize YC Schmitz M Kurt-Jones EA. A novel host-parasite lipid cross-talk. Schistosomal lyso-phosphatidylserine activates toll-like receptor 2 and affects immune polarization. J Biol Chem. (2002) 277:48122–9. 10.1074/jbc.M206941200 12359728

[36] Xiang X Poliakov A Liu C Liu Y Deng ZB Wang J. Induction of myeloid-derived suppressor cells by tumor exosomes. Int J Cancer. (2009) 124:2621–33. 10.1002/ijc.24249 19235923 PMC2757307

[37] Record M Carayon K Poirot M Silvente-Poirot S. Exosomes as new vesicular lipid transporters involved in cell-cell communication and various pathophysiologies. Biochim Biophys Acta. (2014) 1841:108–20. 10.1016/j.bbalip.2013.10.004 24140720

[38] Jethwa SA Leah EJ Zhang Q Bright NA Oxley D Bootman MD. Exosomes bind to autotaxin and act as a physiological delivery mechanism to stimulate LPA receptor signalling in cells. J Cell Sci. (2016) 129:3948–57. 10.1242/jcs.184424 27557622 PMC5087657

[39] Lundy SK Lukacs NW. Chronic schistosome infection leads to modulation of granuloma formation and systemic immune suppression. Front Immunol. (2013) 4:39. 10.3389/fimmu.2013.00039 23429492 PMC3576626

[40] Sombetzki M Koslowski N Rabes A Seneberg S Winkelmann F Fritzsche C. Host defense versus immunosuppression: unisexual infection with male or female *Schistosoma mansoni* differentially impacts the immune response against invading cercariae. Front Immunol. (2018) 9:861. 10.3389/fimmu.2018.00861 29743881 PMC5930291

[41] Mekonnen GG Pearson M Loukas A Sotillo J. Extracellular vesicles from parasitic helminths and their potential utility as vaccines. Expert Rev Vaccines. (2018) 17:197–205. 10.1080/14760584.2018.1431125 29353519

[42] Sotillo J Pearson M Potriquet J Becker L Pickering D Mulvenna J. Extracellular vesicles secreted by Schistosoma mansoni contain protein vaccine candidates. Int J Parasitol. (2016) 46:1–5. 10.1016/j.ijpara.2015.09.002 26460238

[43] Damasceno L Ritter G Batt CA. Process development for production and purification of the *Schistosoma mansoni* Sm14 antigen. Protein Expr Purif. (2017) 134:72–81. 10.1016/j.pep.2017.04.002 28389350

